# Interspinous-Interbody Fusion via a Strictly Lateral Surgical Approach: A Biomechanical Stabilization Comparison to Constructs Requiring Both Lateral and Posterior Approaches

**DOI:** 10.7759/cureus.41918

**Published:** 2023-07-15

**Authors:** Thomas P Hedman, Donna D Ohnmeiss, Jeremi Leasure, Oluwatodimu R Raji, Stephen H Hochschuler

**Affiliations:** 1 Biomedical Engineering, University of Kentucky, Lexington, USA; 2 Research Foundation, Texas Back Institute, Plano, USA; 3 Research, The Taylor Collaboration, San Francisco, USA; 4 Research, The Taylor Foundation, San Francisco, USA; 5 Orthopedic Surgery, Texas Back Institute, Plano, USA

**Keywords:** lateral interbody fusion, pedicle screw, facet screw, interspinous fixation device, lumbar fusion, biomechanics

## Abstract

Objective

Lumbar fusion performed through lateral approaches is becoming more common. The interbody devices are generally supported by supplemental posterior fixation implanted through a posterior approach, potentially requiring a second incision and intraoperative repositioning of the patient. A minimally invasive lateral interspinous fixation device may eliminate the need for intraoperative repositioning and avoid disruption of the supraspinous ligament. The objective of this in vitrobiomechanical study was to investigate segmental multidirectional stability and maintenance of foraminal distraction of a lateral interspinous fixation device compared to commonly used pedicle screw and facet screw posterior fixation constructs when combined with lumbar interbody cages.

Methods

Six human cadaver lumbar spine specimens were subjected to nondestructive quasistatic loading in the following states: (1) intact; (2) interspinous fixation device alone and (3) with lateral interbody cage; (4) lateral lumbar interbody cage with bilateral pedicle screws; (5) lateral lumbar interbody cage with unilateral pedicle screws; and (6) lateral lumbar interbody cage with facet screws. Multidirectional pure bending in 1.5 Nm increments to 7.5 Nm, and 7.5 Nm flexion-extension bending with a 700 N compressive follower load were performed separately with optoelectronic segmental motion measurement. Relative angular motions of L2-L3, L3-L4, and L4-L5 functional spinal units were evaluated, and the mean instantaneous axis of rotation in the sagittal plane was calculated for the index level. Foraminal height was assessed during combined flexion-extension and compression loading for each test construct.

Results

All implant configurations significantly restricted flexion-extension motion compared with intact (p < 0.05). No significant differences were found in flexion-extension when comparing the different posterior implants combined with lateral lumbar interbody cages. All posterior fixation devices provided comparable neuroforaminal distraction and maintained distraction during flexion and extension.

Conclusions

When combinedwith lateral lumbar interbody cages, the minimally invasive lateral interspinous fixation device effectively stabilized the spine and maintained neuroforaminal distraction comparable to pedicle screw constructs or facet screws. These results suggest the lateral interspinous fixation device may provide a favorable alternative to other posterior systems that require patient repositioning during surgery and involve a greater disruption of native tissues.

## Introduction

The primary goal of lumbar fusion is to permanently stabilize the operated segment, typically in combination with a neural decompression procedure to address various neuropathies. As described in a comprehensive review, this stabilization is best achieved with a combination of interbody fusion and posterior fixation [1]. Traditionally, stabilization of these spinal columns was achieved through sequential open surgical approaches, including anterior lumbar interbody fusion and posterior pedicle screws and rods. Many alternatives for interbody fusion have been developed, including several employing transforaminal and lateral approaches. Posterior fixation has broadened to include facet screws and interspinous process devices. While transforaminal interbody cages can be delivered from a posterior approach, the lateral approach allows for a larger cage to cover more of the interbody space [2] as well as greater endplate preparation compared with other interbody techniques [3]. Minimally invasive surgery (MIS) techniques have been developed for implanting posterior fixation devices to further stabilize spinal segments treated with lateral interbody fusion cages. However, these techniques potentially require re-positioning the patient, with the correlative increase in operative time for the positioning and preparation for the posterior procedure. Lateral plates have been introduced to help stabilize the segment without repositioning. These have been found to provide less stability than pedicle screw fixation [4], and there have been reports of a possible increased risk of vertebral body fracture associated with these plates [5,6]. A minimally invasive interspinous fixation device (IFD) was developed that could be placed through a direct lateral approach, thus eliminating the need to reposition the patient after lateral interbody fusion and preventing any disruption of the supraspinous ligament. The purpose of this study was to compare the biomechanical segmental fixation properties of this device to other posterior fixation systems.

Lumbar spinal stenosis is a common condition with approximately 1.4 million new cases diagnosed annually in the US [7]. Surgical intervention for this condition often involves a decompression laminectomy, which unavoidably causes destabilization of the lumbar segment [8]. This iatrogenic destabilization is made worse by surgical disruption of the supraspinous-interspinous ligament complex, which has been shown to contribute more than a third of the resistance to joint flexion [9]. A secondary objective of this study was to compare the ability of the different posterior systems to maintain distraction of the foramen during loading as an indication of their ability to provide indirect foraminal decompression.

## Materials and methods

Specimen selection and preparation

Six cadaver lumbar spines (L1-sacrum) were acquired for testing from an American Association of Tissue Banks accredited tissue bank. The ages ranged from 40 to 80 years; male to female ratio was 1:2. Donors with a history of spine surgery or abdominal surgery were excluded. Specimens were fluoroscopically screened for any signs of deformity or prior spine surgery before inclusion into the study. The cadaver spinal columns were cleaned of musculature and soft tissue with care taken not to disrupt any ligaments connecting the vertebrae. Each specimen was potted at L1 and S1 with epoxy resin.

Device descriptions and techniques

**Figure 1:**
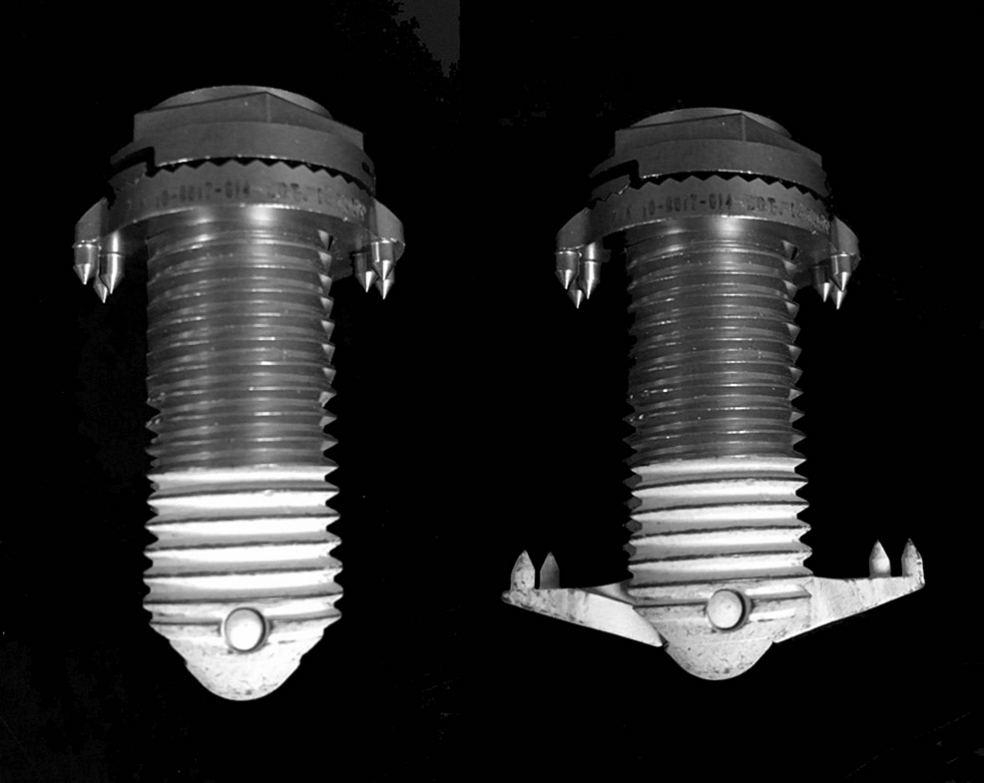
The interspinous fixation device used in the study was designed to be placed from a lateral approach. The extensions, deployed after the device is passed through the interspinous ligament, have teeth to anchor onto the spinous processes.

To accommodate anatomic restrictions of the cadaveric lumbar spines, three sizes of lateral interbody cages (K2M, Leesburg, VA, USA) were used in this study. Pedicle screws of 6.5 mm diameter and titanium alloy fusion rods of 5.5 mm diameter were used for the bi-lateral and uni-lateral pedicle screw constructs in this study, and 4.5 mm facet screws were used for the facet screw constructs. The minimally invasive lateral IFD used for the study was the Minuteman G3 (Spinal Simplicity, Overland Park, KS, USA; Figure 1). It has a threaded cylinder with the capacity to hold bone graft materials. Three diameters of the cylinder, 10 to 14 mm, were used to match the interspinous process distances at the target levels.

Test conditions

**Figure 2:**
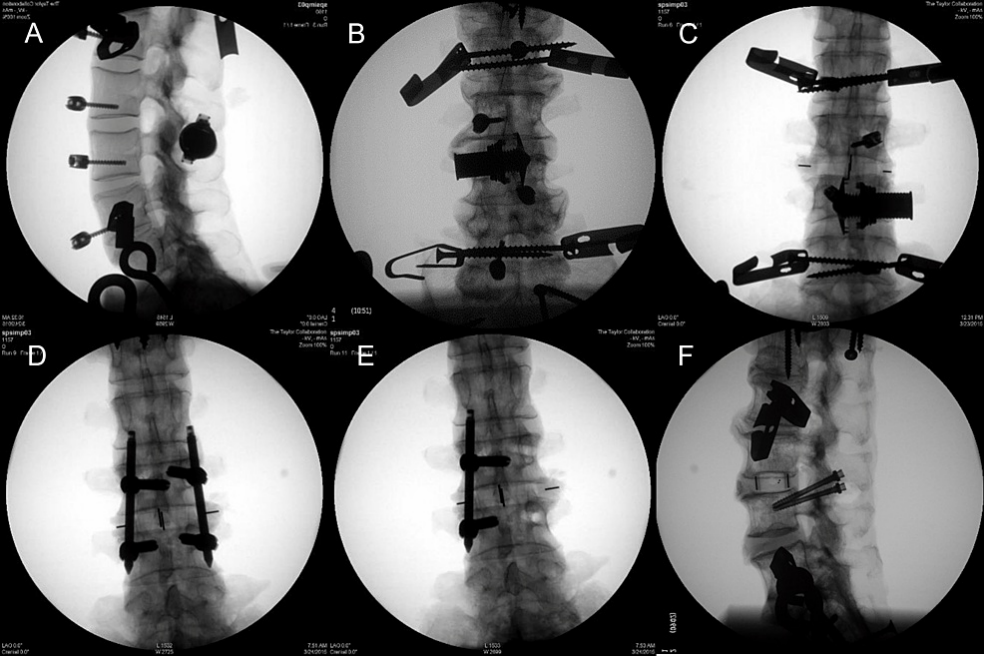
Fluoroscopic images of the instrumented constructs. (A) Interspinous fixation device – lateral view; (B) interspinous fixation device – anterior-posterior view; (C) interspinous fixation device plus lateral interbody cage (radiopaque markers are visible); (D) bilateral pedicle screws and connecting rods plus lateral interbody cage; (E) unilateral pedicle screws and connecting rod plus lateral interbody cage; (F) facet screws plus lateral interbody cage.

Each of the six specimens was tested in six implant configurations, including no fixation devices (INTACT); the second construct was an IFD implanted at L3-4; and the third construct was a direct lateral lumbar interbody fusion (LLIF) cage placed in the L3-4 disc space in addition to the IFD (LLIF+IFD). This group received a unilateral annulotomy and discectomy followed by a contralateral release of the annulus to allow for the distraction of the disc space. The distraction of the neuroforamen for both constructs that included the IFD was achieved by sizing the implant equal to the interspinous distance at the anterior base of the cranial and caudal processes measured on fluoroscopy. To prepare the specimens for this device, a lateral approach rasping of the interspinous ligament and decortication of the cranial and caudal spinous processes was performed. The device was passed through the interspinous ligament, leaving the remaining interspinous ligament and all of the supraspinous ligament intact. After positioning, the extension plates on the distal side were deployed and the device was compressed against the spinous processes of the level being treated. The fourth construct was an LLIF cage in the L3-4 disc space combined with bilateral transpedicular fixation screws at L3 and L4 followed by connecting fusion rods (LLIF+biPS). This group received pilot holes with a standard point awl followed by pedicle screw insertion. No tap was used for pilot hole preparation. Distraction of the neuroforamen was achieved by locking the caudal pedicle screw blockers to the rods, followed by a 2 mm distraction of the cranial screws using a standard distractor instrument. The fifth construct included the interbody cage in the L3-4 disc space in addition to unilateral trans-pedicular fixation screws at L3 and L4 with a connecting fusion rod (LLIF+uniPS). A similar 2 mm distraction was applied to the single fusion rod and screw construct. The sixth construct included the interbody cage in the L3-4 disc space in addition to trans-facet fusion screws between L3 and L4 (LLIF+FS). This group received a pilot hole using a powered drill bit in a caudal-lateral direction from the inferior facet of L3 to the superior facet of L4. Fluoroscopic images of the five instrumented constructs (IFD shown in both anterior-posterior and lateral views) are shown in Figure 2. The order of constructs was maintained across all specimens. The order progressed from the construct with the least to the most amount of specimen tissue alteration (IFD-only had no anterior column component, and facet screws penetrated the facets at the joint level). Validation of interbody cage and posterior instrumentation positions were confirmed using fluoroscopic X-rays for each construct on each spine specimen.

Mechanical testing

Two experimental loading protocols were conducted using an MTS MiniBionix 8521 testing system (MTS Systems, Eden Prairie, MN, USA). In the first protocol, pure bending moments were generated using custom loading fixtures attached to the MTS system. Multi-directional segmental motions were evaluated under flexion, extension, left and right lateral bending, and axial rotation loading. Bending moments were increased in 1.5 Nm increments to 7.5 Nm. A three-dimensional (3D) motion tracking system (Optotrak 3020, NDI, Ontario, Canada) used markers that were attached to L2, L3, L4, and L5 vertebral bodies to track their positions and orientations during loading. Relative angular motions of L2-L3, L3-L4, and L4-L5 functional spinal units were subsequently and concurrently calculated alongside the mean instantaneous axis of rotation in the sagittal plane, which was calculated for the index spinal units between the maximum applied moments.

In the second protocol, 7.5 Nm of flexion and extension bending moments were applied with a 700 N axial compressive follower load in accordance with previous studies [10-13]. Foraminal heights in the neutral position and at maximum flexion and extension loading were calculated at the index level, L3-4, from 3D motion data using the rigid body methods described by Lazaro et al. [12]. A comparison of outcome measures between the device groups was performed using standard one-way ANOVA methods and an alpha equal to 0.05.

## Results

To highlight the important observations from the study, the results presented in this article include the primary motions, instantaneous axis of rotation, and foraminal height outcomes from the index level only. Additional data and results can be obtained from the authors upon request.

Multi-directional range of motion

**Figure 3:**
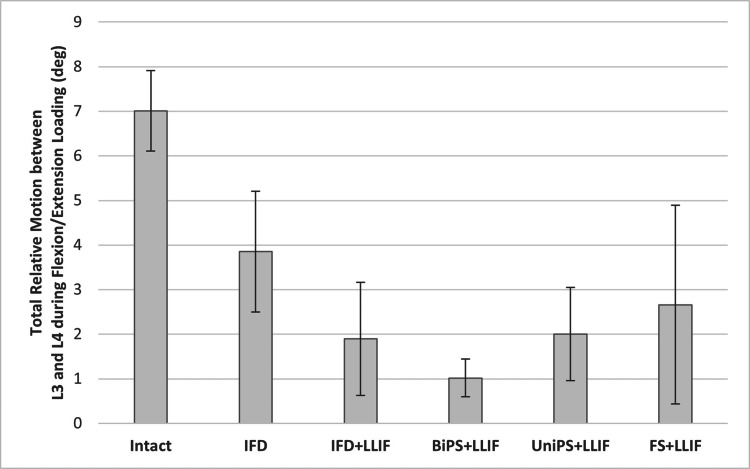
The mean flexion-extension range of motion (degrees) at L3-4 for each test construct compared to INTACT (lines represent standard deviation). The motion was significantly less in all conditions involving an implant compared with INTACT (p < 0.05). IFD: interspinous fixation device; IFD+LLIF: lateral lumbar interbody fusion cage plus interspinous fixation device; BiPS+LLIF: lateral lumbar interbody fusion cage plus bilateral pedicle screws and connecting rods, UniPS+LLIF: lateral lumbar interbody fusion cage plus unilateral pedicle screws and connecting rod; FS+LLIF: lateral lumbar interbody fusion cage plus facet screws.

Mean flexion-extension motion at L3-4 was significantly less in all conditions involving an implant compared with INTACT (p < 0.05; Figure 3 and Table 1). The four conditions involving posterior fixation combined with LLIF (LLIF+IFD, LLIF+biPS, LLIF+uniPS, and LLIF+FS) produced comparable values during flexion-extension bending moments with mean reductions in motion compared to the intact state ranging from 86% (LLIF+biPS) to 61% (LLIF+FS). IFD posterior fixation without an accompanying LLIF reduced flexion-extension motion by 44% compared to INTACT.

**Table 1:** Mean and standard deviation of the motion (degrees) in the three pure bending modes. IFD: interspinous fixation device; LLIF+IFD: lateral lumbar interbody fusion cage plus interspinous fixation device; LLIF+biPS: lateral lumbar interbody fusion cage plus bilateral pedicle screws and connecting rods; LLIF+uniPS: lateral lumbar interbody fusion cage plus unilateral pedicle screws and connecting rod; LLIF+FS: lateral lumbar interbody fusion cage plus facet screws.

Condition	Flexion-extension	Axial rotation	Lateral bending
INTACT	7.0 ± 0.9	4.4 ± 1.8	5.7 ± 1.7
IFD	3.9 ± 1.4	3.7 ± 0.8	4.7 ± 1.9
LLIF+IFD	1.9 ± 1.3	3.6 ± 1.7	3.9 ± 2.4
LLIF+biPS	1.0 ± 0.4	2.7 ± 0.7	1.6 ± 0.6
LLIF+uniPS	2.0 ± 1.0	3.1 ± 0.9	3.3 ± 1.1
LLIF+FS	2.7 ± 2.2	3.5 ± 1.3	3.9 ± 2.7

During axial rotation, the only statistically significant difference was that LLIF+biPS reduced mean axial motion by 39% compared to the intact state (p < 0.05; Table 1). Unlike the results seen in flexion-extension testing, the addition of LLIF had little effect on the reduction of axial motion of the IFD.

In lateral bending, both of the pedicle screw constructs significantly reduced motions compared to INTACT, 72% for LLIF+biPS and 42% for LLIF+uniPS (p < 0.05; Table 1), and LLIF+biPS allowed significantly less motion than the IFD alone (66%, p < 0.05). Similar to flexion-extension results, lateral bending motions for the LLIF+IFD, LLIF+uniPS, and LLIF+FS were comparable with mean values of 3.3 degrees to 3.9 degrees (both LLIF+IFD and LLIF+FS).

Instantaneous axis of rotation (IAR)

**Figure 4:**
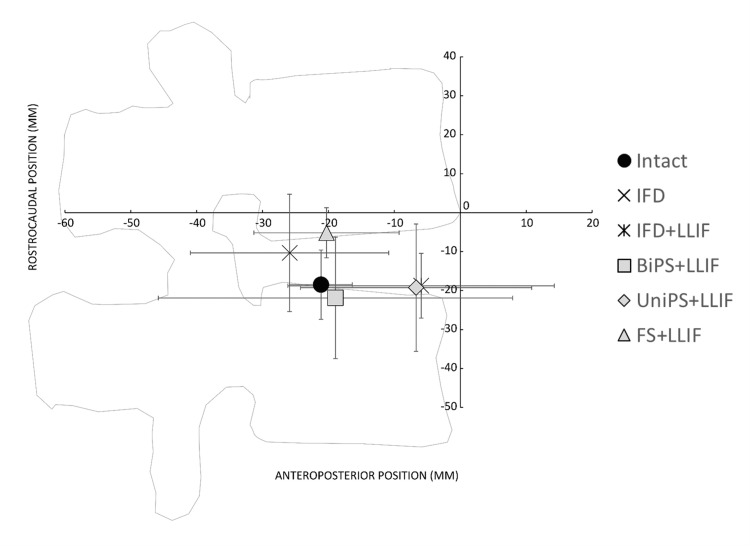
Location of mean instantaneous axis of rotation for INTACT and each treatment condition (lines represent standard deviations along the two axes) during flexion-extension loading. IFD: interspinous fixation device; IFD+LLIF: lateral lumbar interbody fusion cage plus interspinous fixation device; BiPS+LLIF: lateral lumbar interbody fusion cage plus bilateral pedicle screws and connecting rods, UniPS+LLIF: lateral lumbar interbody fusion cage plus unilateral pedicle screws and connecting rod; FS+LLIF: lateral lumbar interbody fusion cage plus facet screws.

There were no statistically significant differences in the anterior-posterior location of the mean IAR when comparing surgical constructs (Figure 4 and Table 2). In the cranial-caudal direction, the mean IAR for LLIF+FS was significantly higher (indicating more cranial position), than LLIF+biPS (p < 0.05).

**Table 2:** Mean instantaneous axis of rotation in the sagittal plane of the index level at the maximum applied moments. Values are mean + standard deviation, and units are mm. The only statistically significant difference was the location for the LLIF+FS construct being significantly more cranial than LLIF+biPS (p < 0.05). IFD: interspinous fixation device; LLIF+IFD: lateral lumbar interbody fusion cage plus interspinous fixation device; LLIF+biPS: lateral lumbar interbody fusion cage plus bilateral pedicle screws and connecting rods; LLIF+uniPS: lateral lumbar interbody fusion cage plus unilateral pedicle screws and connecting rod; LLIF+FS: lateral lumbar interbody fusion cage plus facet screws.

Condition	Anterior-posterior	Cranial-caudal
INTACT	-21.1 ± 4.8	-18.5 ± 8.9
IFD	-25.9 ± 15.0	-10.3 ± 16.0
LLIF+IFD	-5.9 ± 20.2	-18.7 ± 8.3
LLIF+biPS	-19.0 ± 26.9	-21.9 ± 15.6
LLIF+uniPS	-6.7 ± 17.5	-19.2 ± 16.4
LLIF+FS	-20.3 ± 11.0	-5.2 ± 6.4

Foraminal height

There were no statistically significant differences in foraminal height between any of the constructs when compared to the INTACT specimen or each other (Table 3). There were also no significant differences in foraminal height changes during flexion or extension loading; however, the INTACT and LLIF+uniPS construct foraminal heights decreased a small amount (<1.2 mm) on average during flexion and extension, while the foraminal heights of the other constructs all increased a small amount (<0.9 mm) on average during flexion and extension.

**Table 3:** Foraminal height at the index level in test conditions of unloaded, and compression loaded in neutral, extension, and flexion (values are mean +/- standard deviation; units are mm). IFD: interspinous fixation device; LLIF+IFD: lateral lumbar interbody fusion cage plus interspinous fixation device; LLIF+biPS: lateral lumbar interbody fusion cage plus bilateral pedicle screws and connecting rods; LLIF+uniPS: lateral lumbar interbody fusion cage plus unilateral pedicle screws and connecting rod; LLIF+FS: lateral lumbar interbody fusion cage plus facet screws.

Condition	Unloaded	Neutral	Extension	Flexion
Intact	17.7 + 3.8	17.8 + 4.0	16.7 + 3.8	17.2 + 4.3
IFD	19.3 + 4.3	19.4 + 2.9	19.7 + 4.1	19.5 + 3.9
LLIF+IFD	20.9 + 4.3	19.4 + 4.9	19.6 + 4.9	19.6 + 4.3
LLIF+biPS	21.1 + 4.4	18.7 + 4.4	20.15 + 4.5	20.3 + 3.9
LLIF+uniPS	19.9+ + 4.3	19.1 + 4.5	18.94 + 4.4	18.7 + 4.7
LLIF+FS	19.9 + 4.4	18.3 + 4.9	18.46 + 5.1	18.6 + 4.6

## Discussion

In this biomechanical human cadaveric study, a minimally invasive lateral IFD combined with a lateral interbody cage was found to provide segmental spinal stability comparable to that of other constructs employing pedicle screws or facet screws combined with lateral interbody cages. It was also found that foraminal distraction using this interspinous fixation technique was similar to commonly used posterior constructs and that foraminal height was maintained during motion under combined compression with flexion-extension bending moments.

The results of the current study are in line with previous investigations; when used with an interbody fusion cage, interspinous fixation produces similar and even superior stability compared to adding pedicle or facet fixation [14-18]. Fogel et al. found the interspinous-interbody combination performed comparable to bilateral pedicle screws supplementing an interbody cage [14]. Wang et al. reported superior stability with the interspinous-interbody combination compared to both facet and pedicle fixation [15]. In other studies that investigated the effects of supplemental fixation after lateral fusion cage placement, it was reported that interspinous devices provide flexion, extension, and axial rotation stability similar to bilateral pedicle screws [16], or favorable stability in flexion-extension with lesser stability in lateral bending or axial rotation [17]. An interspinous fusion device combined with anterior lumbar interbody fusion (ALIF) provided stability similar to ALIF supplemented with bilateral pedicle screws and rods and greater stability than an anterior plate [18]. In a review article, Hardenbrook et al. found that biomechanical data suggested that IFDs provided spinal stability similar to that achieved with pedicle screws [19]. He added that the IFDs appear to provide a less invasive option to pedicle or facet screws while achieving similar biomechanical stabilization. He also made an important differentiation between spinous process rigid fixation and spinous process dynamic spacers, which provide limited dynamic motion at the joint. Likewise, it is important to note the results of the present study do not apply to dynamic spacers or the use of dynamic spacers with lateral interbody fusion.

Few biomechanical studies have investigated the ability of interspinous fixation devices to distract the neuroforamen. Lazaro et al. [12] investigated foraminal height changes with a dynamic interspinous spacer. They found that the spacer reduced loss of foraminal height compared to intact when the spine specimen was loaded in extension. Interspinous spacers do not restrict flexion motion, so these results only corroborate the current findings of IFD maintaining foraminal distraction in extension. One cadaveric study with compressive follower preloads found that lateral interbody fusion supplemented with pedicle screws was associated with significantly greater foraminal area than with interbody fusion and lateral plating [20]. While interbody fusion may increase foraminal height, there is concern about subsidence with a potential for a reduction in foraminal height. In a review including lateral interbody fusion, Joseph et al. found a 10.8% overall rate of cage subsidence in patients [21]. Another study reported subsidence of at least 25% in more than half of the patients treated with a stand-alone interbody cage implanted using a lateral approach [22]. Results of another clinical study reported that stand-alone interbody fusion performed using an extreme lateral approach significantly increased foraminal height, foraminal area, and central canal diameter [23]. However, 10% of patients underwent subsequent surgery involving adding pedicle screws at the index level for distraction, and the authors did warn about the possible reduction of indirect decompression due to the subsidence of stand-alone cages. Posterior fixation may be a means of reducing the risk of subsidence.

The results of the current study found that the laterally placed interspinous fusion device combined with a lateral interbody fusion cage provided multi-directional segmental stability and maintenance of neuroforaminal distraction during flexion-extension loading similar to pedicle screws or facet screws used with lateral interbody fusion cages. Thus, from a biomechanical standpoint, the laterally placed interspinous fixation device is a viable alternative to currently used posterior fixation devices used with lateral interbody cages. The lateral IFD implant in this investigation retains the supraspinous ligament, which may increase stabilization as compared to other interspinous devices that remove or otherwise disrupt this ligament during placement. Likewise, there is penetration through a portion of the interspinous ligament with the IFD procedure rather than the larger disruption of this ligament during implantation of other minimally invasive interspinous devices. The benefit of the preservation of these ligaments for joint stabilization appears to be supported by a biomechanical study that suggests that the supraspinous-interspinous ligament complex contributes more than a third of the resistance to flexion in the intact joint [9].

Limitations of this study include using a cadaveric model, which undergoes ambient degradation between the rounds of testing that is unlikely to be observed in an in vivo study. The number of human cadaveric spine specimens was also a limitation of this study. While using a much larger number of specimens would enable minimization of environmental degradation and minimize or quantify prior-loading degradation (intra-specimen variability), the use of the study design employed in this study - testing each construct on each spine specimen - reduces the amount of inter-specimen variability in the data and significantly reduces the number of human spine specimens required to gather an equal number of data points. The loading was non-destructive and the specimens were kept moist; however, performing successive tests on the same specimen could result in lower apparent stability in the later tests performed. The experiments were conducted on lumbar spines with a male-to-female ratio of 1:2 and ages ranging from 40 to 80 years. Therefore, it may not be appropriate to generalize the results from this study to different demographics or patients with varying initial spinal conditions. Additional studies are required to determine if the joint stabilization demonstrated in the in vitro environment will correspond to comparable stabilization and satisfactory levels of bony fusion occurring in vivo. While maintenance of foraminal height under combined compressive and flexion-extension loading was demonstrated in this study, increases and maintenance of neuroforaminal area and canal area were not assessed, but would be relevant in evaluating the clinical utility of posterior fixation devices used without direct neural decompression, in particular, the lateral IFD, in spinal stenosis cases. Although the subsequent addition of posterior instrumentation is common practice in cases of subsidence or other complications related to stand-alone lateral interbody cages, the competence of posterior fixation constructs in combination with LLIF cages to resist cage subsidence cannot be ascertained from these biomechanical study data. Finally, a clinical investigation is warranted to determine if the IFD fulfills the potential to reduce operative time and the need for additional incisions by avoiding the placement of supplemental fixation from a posterior approach following the placement of an interbody fusion cage from a lateral approach.

## Conclusions

When combined with LLIF, the minimally invasive lateral interspinous fixation device effectively stabilized the spine and maintained neuroforaminal distraction comparable to pedicle screws and rods or facet screws. These results suggest the lateral interspinous fixation device may provide a favorable alternative to other posterior fixation systems that require patient repositioning during surgery and involve a greater disruption of native tissues. Clinical investigations are needed to determine the benefits of lateral interspinous fixation combined with lateral interbody fusion with regard to operating time, rates of subsidence, levels of bony fusion, and other clinical outcomes.

## References

[1] Oxland TR, Lund T (2000). Biomechanics of stand-alone cages and cages in combination with posterior fixation: a literature review. Eur Spine J.

[2] Pimenta L, Turner AW, Dooley ZA, Parikh RD, Peterson MD (2012). Biomechanics of lateral interbody spacers: going wider for going stiffer. ScientificWorldJournal.

[3] Tatsumi R, Lee YP, Khajavi K, Taylor W, Chen F, Bae H (2015). In vitro comparison of endplate preparation between four mini-open interbody fusion approaches. Eur Spine J.

[4] Kim SM, Lim TJ, Paterno J, Park J, Kim DH (2005). Biomechanical comparison: stability of lateral-approach anterior lumbar interbody fusion and lateral fixation compared with anterior-approach anterior lumbar interbody fusion and posterior fixation in the lower lumbar spine. J Neurosurg Spine.

[5] Tender GC (2014). Caudal vertebral body fractures following lateral interbody fusion in nonosteoporotic patients. Ochsner J.

[6] Le TV, Smith DA, Greenberg MS, Dakwar E, Baaj AA, Uribe JS (2012). Complications of lateral plating in the minimally invasive lateral transpsoas approach. J Neurosurg Spine.

[7] Kalichman L, Cole R, Kim DH, Li L, Suri P, Guermazi A, Hunter DJ (2009). Spinal stenosis prevalence and association with symptoms: the Framingham Study. Spine J.

[8] Smith ZA, Vastardis GA, Carandang G (2014). Biomechanical effects of a unilateral approach to minimally invasive lumbar decompression. PLoS One.

[9] Gillespie KA, Dickey JP (2004). Biomechanical role of lumbar spine ligaments in flexion and extension: determination using a parallel linkage robot and a porcine model. Spine (Phila Pa 1976).

[10] Lindsey DP, Swanson KE, Fuchs P, Hsu KY, Zucherman JF, Yerby SA (2003). The effects of an interspinous implant on the kinematics of the instrumented and adjacent levels in the lumbar spine. Spine (Phila Pa 1976).

[11] Swanson KE, Lindsey DP, Hsu KY, Zucherman JF, Yerby SA (2003). The effects of an interspinous implant on intervertebral disc pressures. Spine (Phila Pa 1976).

[12] Lazaro BC, Brasiliense LB, Sawa AG, Reyes PM, Theodore N, Sonntag VK, Crawford NR (2010). Biomechanics of a novel minimally invasive lumbar interspinous spacer: effects on kinematics, facet loads, and foramen height. Neurosurgery.

[13] Wilson DC, Niosi CA, Zhu QA, Oxland TR, Wilson DR (2006). Accuracy and repeatability of a new method for measuring facet loads in the lumbar spine. J Biomech.

[14] Fogel GR, Parikh RD, Ryu SI, Turner AW (2014). Biomechanics of lateral lumbar interbody fusion constructs with lateral and posterior plate fixation: laboratory investigation. J Neurosurg Spine.

[15] Wang JC, Spenciner D, Robinson JC (2006). SPIRE spinous process stabilization plate: biomechanical evaluation of a novel technology. Invited submission from the Joint Section Meeting on Disorders of the Spine and Peripheral Nerves, March 2005. J Neurosurg Spine.

[16] Doulgeris JJ, Aghayev K, Gonzalez-Blohm SA, Lee WE 3rd, Vrionis FD (2015). Biomechanical comparison of an interspinous fusion device and bilateral pedicle screw system as additional fixation for lateral lumbar interbody fusion. Clin Biomech (Bristol, Avon).

[17] Reis MT, Reyes PM, Bse Bse (2016). Biomechanical evaluation of lateral lumbar interbody fusion with secondary augmentation. J Neurosurg Spine.

[18] Karahalios DG, Kaibara T, Porter RW (2010). Biomechanics of a lumbar interspinous anchor with anterior lumbar interbody fusion. J Neurosurg Spine.

[19] Hardenbrook M, Henn JS, Oppenheim J, Shah MV (2013). Spinous process fixation devices for instrumented spinal fusion. Surg Technol Int.

[20] Marulanda GA, Nayak A, Murtagh R, Santoni BG, Billys JB, Castellvi AE (2014). A cadaveric radiographic analysis on the effect of extreme lateral interbody fusion cage placement with supplementary internal fixation on indirect spine decompression. J Spinal Disord Tech.

[21] Joseph JR, Smith BW, La Marca F, Park P (2015). Comparison of complication rates of minimally invasive transforaminal lumbar interbody fusion and lateral lumbar interbody fusion: a systematic review of the literature. Neurosurg Focus.

[22] Marchi L, Abdala N, Oliveira L, Amaral R, Coutinho E, Pimenta L (2013). Radiographic and clinical evaluation of cage subsidence after stand-alone lateral interbody fusion. J Neurosurg Spine.

[23] Oliveira L, Marchi L, Coutinho E, Pimenta L (2010). A radiographic assessment of the ability of the extreme lateral interbody fusion procedure to indirectly decompress the neural elements. Spine (Phila Pa 1976).

